# Rare presentation of retroperitoneal leiomyosarcoma mimicking a myoma in a 46‐year‐old woman: A case report

**DOI:** 10.1002/ccr3.6909

**Published:** 2023-01-28

**Authors:** Setareh Akhavan, Shahrzad Sheikhhasani, Mohades Peydayesh, Shima Alizadeh, Fatemeh Zamani, Narges Zamani

**Affiliations:** ^1^ Department of Oncologic Gynecology, Vali‐e‐Asr Hospital Tehran University of Medical Science Tehran Iran; ^2^ Department of Gynecology Pasteur Hospital, Bam University of Medical Science Bam Iran; ^3^ Department of Obstetrics and Gynecology, Vali‐e‐Asr Hospital Tehran University of Medical Sciences Tehran Iran; ^4^ Department of Radiology, Children Medical Centre of Excellence Tehran University of Medical Science Tehran Iran

**Keywords:** internal iliac vein, leiomyosarcoma, retroperitoneal space, surgical resection

## Abstract

Retroperitoneal sarcoma is relatively uncommon. We share our experience in encountering retroperitoneal sarcoma with vascular and urethral adhesion in a 46‐year‐old woman. Given the rarity of these tumors, evaluation and management should ideally be performed in a center equipped with multidisciplinary expertise in treating sarcomas.

## 1 INTRODUCTION

Leiomyosarcoma (LMS), a subset of soft tissue sarcomas, refers to malignant smooth muscle neoplasms accounting for about 5%–10% of all sarcomas. Natural history depends on the anatomic location where they arise. About 50% arise in the retroperitoneum/abdomen, including the visceral, uterine, and retroperitoneal, with the uterus being the most common location. Given these tumors' enormous size and frequent location, abdominal pain, nausea, vomiting, anorexia, weight loss, fatigue, and malaise are their most common symptoms.^1^ Retroperitoneal sarcoma (RPS) is relatively uncommon, accounting only for 10%–15% of all soft tissue sarcomas.^2^ In a population‐based series from the Surveillance, Epidemiology, and End Results (SEER) database, the average annual prevalence of RPS was approximately 2.7 cases per one million population.^3^ The LMS of the retroperitoneum arises from the inferior vena cava, its tributaries, or any small vessel. When diagnosed, they often emerge as a mass, usually in enormous size. Most knowledge in treating inferior vena cava (IVC) LMS comes from case reports and case series. Surgical resection offers the best chance of long‐term survival and the likelihood of a cure. Remarkably, the five‐year disease‐free survival rate of patients undergoing resection was between 30% and 60%; however, the survival in those who did not undergo surgery was often below 1 year.^4^, ^5^, ^6^, ^7^, ^8^ We aimed to share our experience in encountering RPS with vascular and ureteral adhesion in the operation room about a patient scheduled for surgery as a case of leiomyoma.

## 2 CASE PRESENTATION

**FIGURE 1:**
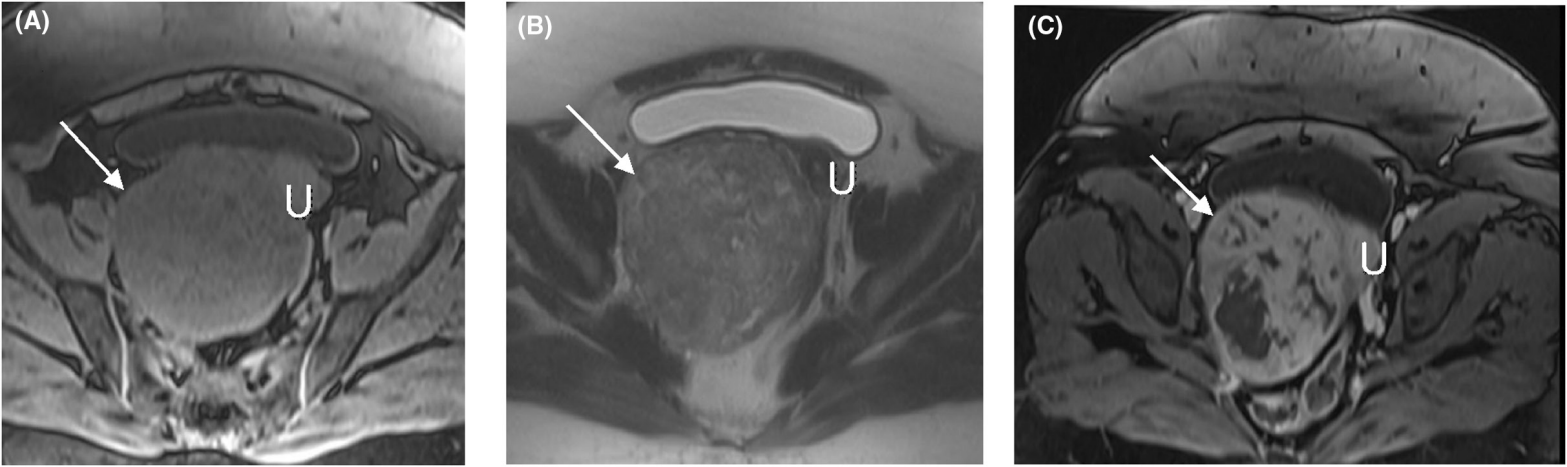
Axial T1‐weighted fat‐suppressed (A), T2‐weighted (B), and gadolinium‐enhanced T1‐weighted fat‐suppressed (C) MR images show a large relatively well‐defined, homogeneous, non‐fat‐containing solid intra‐abdominal mass that is isointense to skeletal muscle on T1‐weighted images (arrows on A), heterogeneously T2 hyperintense (arrows on B), and heterogeneously enhanced (arrows on C). Uterus = U on A–C.

**FIGURE 2:**
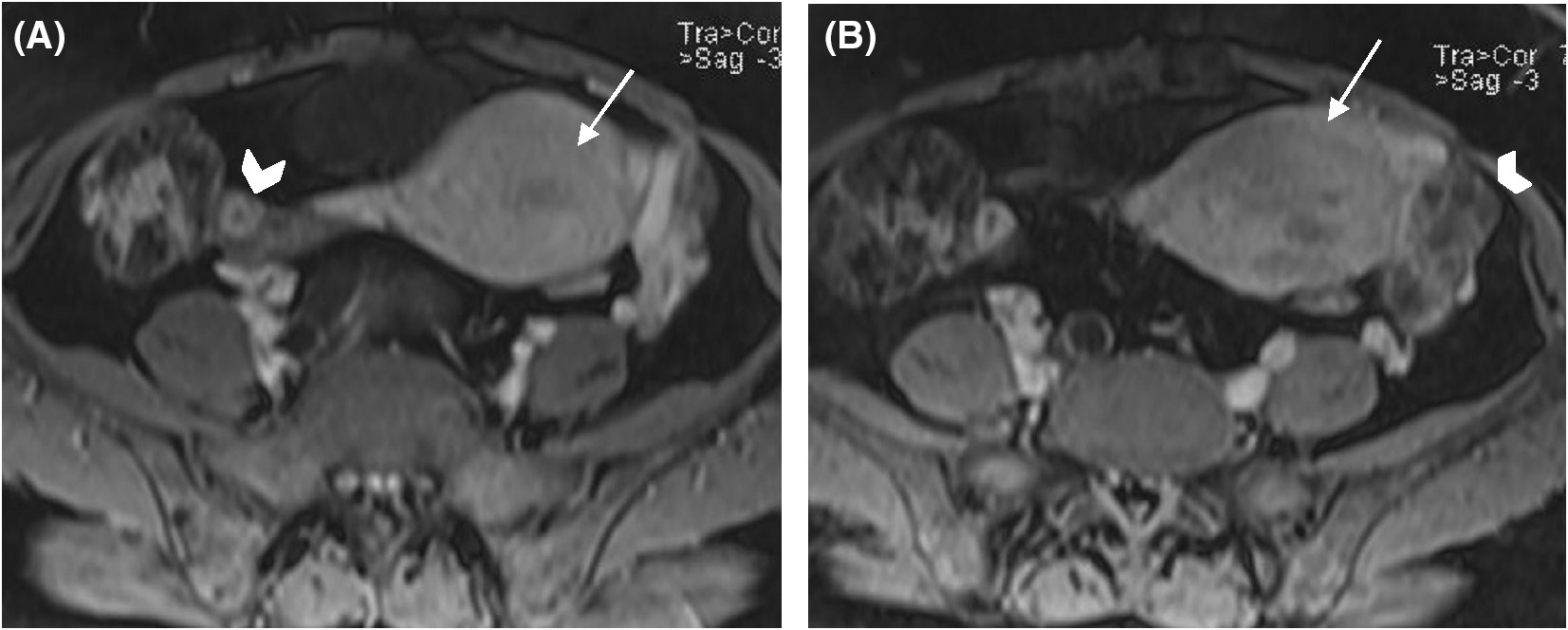
Axial gadolinium‐enhanced T1‐weighted fat‐suppressed (A, B) manifest normal uterus (arrow on A, B) and normal ovaries (arrow head on A, B).

**FIGURE 3:**
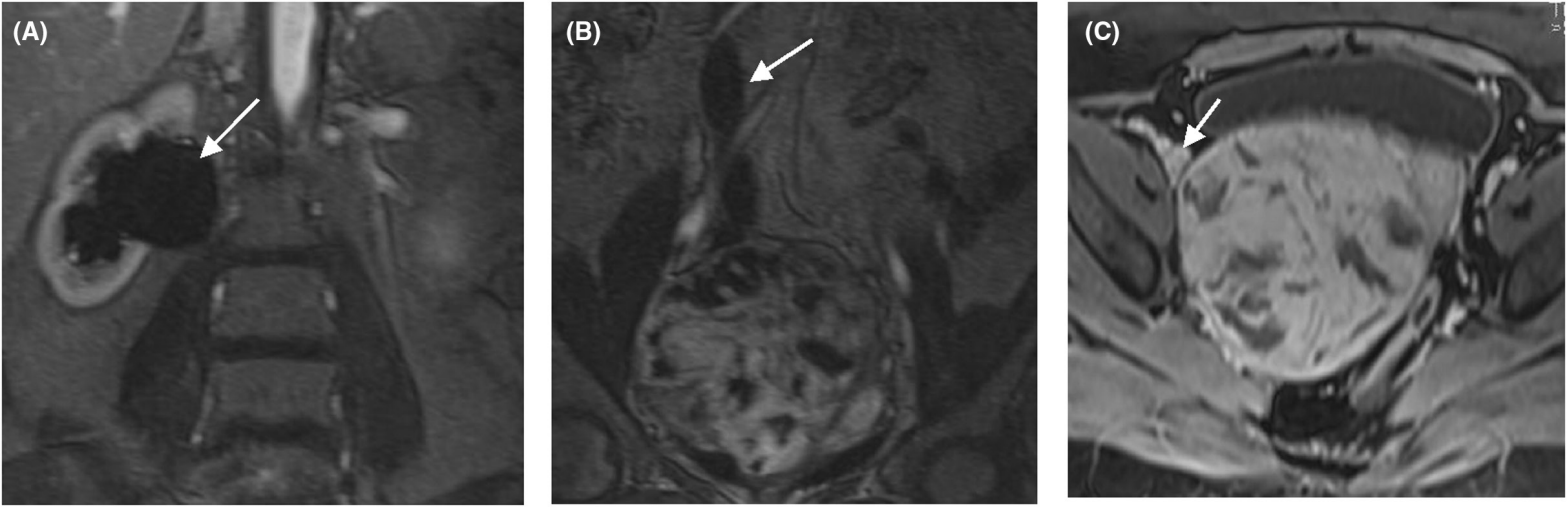
Coronal and axial gadolinium‐enhanced T1‐weighted fat‐suppressed (A–C) MR images show right side hydroureteronephrosis (arrow on A and B) and right external iliac vein (arrow on C).

A 46‐year‐old Gravida 3 para 3 woman with no medical, allergy, or drug use history and with a history of abdominal pain, hypermenorrhea, and pelvic pressure was referred to the gynecology and oncology ward of a university Hospital a few months ago. In the vaginal examination, there was bulging on the right side, which completely deviated the cervix to the left and upward position. On abdominal sonography, the uterus size was 63 × 42 mm with an endometrial thickness of 9 mm. A heterogeneous 114 × 99‐mm mass in a posterior cul‐de‐sac, probably with the uterine origin, was detected. Magnetic resonance imaging (MRI) revealed a well‐defined heterogeneous soft‐tissue mass from degeneration and necrosis in the pelvic cavity posterior to the uterus was observed and separated from the uterus and ovaries. In post‐contrast images, heterogeneous enhancement at the tumor was noticed (Figures 1 and 2). This mass lesion posed pressure on the right ureter, causing hydroureteronephrosis, and was in close contact with the right external iliac vein (Figure 3). She had a surgical history of cesarean section with *Pfannenstiel incision* three times, and her last child was 10 years old. The attempts for ureteral stent placement before surgery had failed; hence, she was scheduled for surgery. The surgery was performed by the fellows of gynecologic oncology under the direct supervision of their attending surgeon. She underwent an exploratory laparotomy under general anesthesia by a midline incision on the suspicion of leiomyoma. A huge solid retroperitoneal mass (16 × 12 × 11 cm) was detected in the right broad ligament, which was attached to the pelvic floor and seemed to be separated from the uterus. Surgical excision aimed to resect the tumor completely. Because of the dense adhesion of the tumor to the ureter and vessels, the internal iliac vein and ureteral injury were torn during dissection. The internal iliac vein was repaired by a vascular surgeon. The intraoperative frozen section analysis suggested a smooth muscle tumor without determining whether it is being benign or malignant. Hysterectomy and bilateral salpingectomy were performed, and the ovaries were preserved.

An internal ureteral Double J‐stent (DJ stent) was inserted, and the primary ureteral was repaired by the urologist. After consulting with an anesthesiologist, we transfused three units of Pack Cell to the patient during the operation due to blood loss. The oral diet began 1 day after surgery, and the patient was discharged 72 h after admission. Before surgery, her hemoglobin level was 10.5 g/dL, and the patient was discharged with Hemoglobin of 9.8 g/dL. Ureteral DJ was removed 6 weeks later with no complication. Pathologic examination revealed grade II/III LMS with a mitotic rate of 12/10 HPF, including atypical forms. The necrosis extent was <50%, and the margin could not be assessed. The pathologic stage was pT4. The patient was candidate for radiation; hence, external beam radiotherapy was provided for her for 25 sessions.

## 3 DISCUSSION

Retroperitoneal sarcoma are rare and account for 10%–15% of all soft tissue sarcomas.^2^ Most tumors are already large and locally advanced when they are first detected (median size at diagnosis is about 15 cm).^9^ Rarely may patients with rapidly expanding high‐grade tumors experience flu‐like symptoms and present with fevers and leukocytosis.^10^, ^11^ Radiographic imaging is a key component of evaluating a patient with a retroperitoneal mass. The preferred diagnostic examination is a contrast‐enhanced computed tomography (CT) scan of the abdomen and pelvis to evaluate the primary site. MRI with gadolinium is reserved for patients with an allergy to iodinated contrast agents or with an equivocal muscle or bone involvement on CT. MRI may also be useful for delineating diseases in the pelvis. For patients with preoperative radiation therapy, MRI is useful to assess the local tumor extent and surrounding edema, which are optimally included in the treatment volume.^12^ In this case, we assumed both sonography and MRI modality. Ultrasound reports revealed a large myoma; however, MRI revealed a well‐defined heterogeneous soft‐tissue mass, which seemed to be malignant; however, it could not specify the origin. The retroperitoneum is the space posterior to the peritoneal cavity and anterior to the para‐spinous musculature.^13^ The structures of the retro peritoneum include the kidneys, adrenal glands, and bilateral perirenal fat, the aorta, and its major branches (e.g., renal arteries), the inferior vena cava and its major tributaries (e.g., renal veins), and the bilateral iliac vessels (namely common, internal, external arteries/veins), the duodenum, and the pancreas. In our case, there was a mass originated in this space, probably from an iliac vein with adhesions to the ureter. Needless to mention that there are several vital structures in the retroperitoneal space, and in our patient, because of the history of three cesarean sections, besides the existence of this mass, accessing the plane for dissection was abstruse. Determining respectability largely depends on the extension of the tumor and the surrounding structures it involves. A relative contraindication is a vascular involvement because reconstructive options vary, given tumor location and tissue involvement.^14^ We confronted a retroperitoneal mass instead of uterus leiomyoma, and supposing RPS, the ability to perform a complete surgical resection at the time of initial presentation, is the most important prognostic factor for survival.^10^, ^11^, ^15^, ^16^, ^17^, ^18^, ^19^, ^20^ Our patient was a married 46‐year‐old woman, and we decided to have complete resection for her survival. Extensive vascular involvement (aorta, vena cava, and/or iliac vessels), although involvement of the vena cava and iliac veins is a relative, rather than absolute, contraindication for respectability, as these vessels can often be ligated or replaced with interposition grafts.^21^ Despite distorted anatomy, we dissected the tumor through adhesions from adjacent structures. Managing bulky intra‐abdominal extra‐luminal tumors is challenging due to their proximity or diffusion of other structures. The surgical resection of retroperitoneal leiomyosarcoma (RPLMS) can be associated with significant morbidity, given that they usually invade main vascular structures such as the inferior vena cava (IVC) and tributaries, the duodenum, and the ureter.^22^ Management needs to be provided by a specialized team of surgeons. Complete resection often needs the extended dissection of the vascular structures, kidneys, bladder, and gastrointestinal tract.^2^ In our experience, we had Urethral Injury that Primary repaired, and DJ placement was done with the cooperation of a urologist. The main challenge was the right internal iliac vein torn during the dissection of the mass. The vascular surgeon repaired the vein. The blood loss estimation made us and the anesthesiology team decide to have the transfusion of Pack‐cell during operation.

The patient underwent Hysterectomy and bilateral salpingectomy. She had a fine post‐operation course without acute complications. The permanent pathology report showed LMS; however, the margin could not be assessed. No pathological finding was reported on the uterus, cervix, and fallopian tubes. Finally, she was a candidate for radiation. van Doorn et al.^23^ reported postoperative high‐dose radiation therapy in 13 out of 34 RPS patients with a significantly decreased recurrence rate. However, the benefits and the effectiveness of radiation therapy have not been rigorously investigated due to the scarcity of cases. Our patient underwent 25 sessions of external radiotherapy. Any discussion on this case would be incomplete without commenting on the ethical aspects of the consent process. she declared that she would be happy if the other doctors and medical students learned from the case and did not mind her condition being discussed. After 6 months of treatment, her health status is favorable, and there is no recurrence or metastasis.

The COVID‐19 pandemic has increased the complexity of cancer care. In this regard, the main issues are balancing the risk of delaying cancer treatment versus COVID‐19 damage, minimizing the number of clinics and hospital visits to reduce exposure, mitigating the adverse effects of social distancing on care delivery, and allocating limited healthcare resources appropriately and fairly.

Retroperitoneal sarcoma is relatively uncommon and difficult to diagnose. Surgical resection is the best treatment for retroperitoneal sarcoma but can be associated with significant morbidity due to their proximity or diffusion of other structures. Because of the rarity of these tumors and the complexity of treatment, evaluation and management should ideally be carried out in centers equipped with multidisciplinary expertise in treating sarcomas in a multidisciplinary tumor board.

## AUTHOR CONTRIBUTIONS

**Setareh Akhavan**: study concept or design, Investigation performed the surgery. **Shahrzad Sheikhhasani**: performed the surgery. **Mohades Peydayesh**: performed the surgery. **Shima Alizadeh**: Writing. **Fatemeh Zamani**: data collection, Imaging determination. **Narges Zamani**: Writing – Review & Editing; critical revision, study concept, performed the surgery.

## CONFLICT OF INTEREST STATEMENT

None.

## ETHICAL APPROVAL

None.

## CONSENT

Written informed consent was obtained from the patient to publish this report in accordance with the journal's patient consent policy. A copy of the written consent is available for review by the Editor‐in‐Chief of this journal on request.

## Data Availability

Data are available for review by the Editor‐in‐Chief of this journal on request.
